# Small Molecular TRAIL Inducer ONC201 Induces Death in Lung Cancer Cells: A Preclinical Study

**DOI:** 10.1371/journal.pone.0162133

**Published:** 2016-09-14

**Authors:** Yuan Feng, Jihong Zhou, Zhanhua Li, Ying Jiang, Ying Zhou

**Affiliations:** 1 Department of Respiratory Medicine, Guangxi University of Traditional Chinese Medicine Affiliated Ruikang Hospital, NanNing, China; 2 Department of Neurology, Guangxi University of Traditional Chinese Medicine Affiliated Ruikang Hospital, NanNing, China; 3 Department of Oncology, Guangxi University of Traditional Chinese Medicine Affiliated Ruikang Hospital, NanNing, China; Institute of Biochemistry and Biotechnology, TAIWAN

## Abstract

Tumor necrosis factor (TNF)-related apoptosis-inducing ligand (TRAIL) selectively targets cancer cells. The present preclinical study investigated the anti-cancer efficiency of ONC201, a first-in-class small molecule TRAIL inducer, in lung cancer cells. We showed that ONC201 was cytotoxic and anti-proliferative in both established (A549 and H460 lines) and primary human lung cancer cells. It was yet non-cytotoxic to normal lung epithelial cells. Further, ONC201 induced exogenous apoptosis activation in lung cancer cells, which was evidenced by TRAIL/death receptor-5 (DR5) induction and caspase-8 activation. The caspase-8 inhibitor or TRAIL/DR5 siRNA knockdown alleviated ONC201’s cytotoxicity against lung cancer cells. Molecularly, ONC201 in-activated Akt-S6K1 and Erk signalings in lung cancer cells, causing Foxo3a nuclear translocation. For the *in vivo* studies, intraperitoneal injection of ONC201 at well-tolerated doses significantly inhibited xenografted A549 tumor growth in severe combined immunodeficient (SCID) mice. Further, ONC201 administration induced TRAIL/DR5 expression, yet inactivated Akt-S6K1 and Erk in tumor tissues. These results of the study demonstrates the potent anti-lung cancer activity by ONC201.

## Introduction

Global cancer studies show that lung cancer causes over one million mortalities each year [1,2,3]. Its incidence has been rising over past decades [1,2,3]. Over 80% of all lung cancers are non-small cell lung cancer (NSCLC) [1,2,3]. Current treatment options for lung cancer include surgical resection, platinum-based chemotherapy, and radiation therapy [4,5]. Unfortunately, the response of these traditional therapies has been far from satisfactory [4,5]. Consequently, lung cancer is rarely curable and prognosis is often poor, with a 5-year overall survival less than 15% [4,5].

Immune checkpoint proteins (or checkpoints) are important inhibitory immune signalings [6]. Existing evidences have shown that inhibition of immune checkpoints, *i*.*e*. using programmed death 1 (PD-1) antibody, could potently provoke therapeutic anti-cancer immunity, which has become a promising anti-lung cancer strategy [6,7]. Some of these checkpoint antibodies have been approved by USA Food and Drug Administration (FDA) for the treatment of lung cancer [8,9]. Yet, depending on the profile of each particular cancer, the response of these antibodies could vary from patient to patient [8,9]. Therefore, there is an urgent need to develop novel and efficient anti-lung cancer agents [4,5].

Tumor necrosis factor (TNF)-related apoptosis-inducing ligand (TRAIL) selectively targets cancer cells while sparing normal cells [10]. Recombinant TRAIL and agonistic anti-TRAIL receptor antibodies are in clinical trials to fight cancer cells [10]. It has become a promising therapeutic option against human cancers [10]. Yet, the clinical use TRAIL or TRAIL-related agents has been limited due to several key drawbacks, including their the short half-life and lack of efficiency [10].

ONC201 is a first-in-class small-molecule TRAIL inducer, which has displayed potent antitumor efficacy *in vitro* and *in vivo* [11,12,13,14]. Previous studies have shown that ONC201 induced TRAIL-mediated apoptosis in several tumor cancer cells [15,16]. At the molecular level, ONC201 was shown to block Akt and Erk signalings, which led to Foxo3a nuclear translocation and transcription of TRAIL and death receptor-5 (DR5) [15,16,17]. To our best knowledge, its potential function in lung cancer cells has not been studied. Here, we performed this preclinical study to investigate the potential anti-cancer efficiency of ONC201 in lung cancer cells.

## Materials and Methods

### 2.1 Chemicals and reagents

ONC201 (TIC10) was obtained from Selleck (Shanghai, China); The pan caspase inhibitor z-VAD-fmk and the caspase-8 inhibitor z-IETD-fmk were from CalBiochem (La Jolla, CA). The kinase antibodies utilized in this study were purchased from Cell Signaling Tech (Shanghai, China). Other antibodies were obtained from Santa Cruz Biotechnology (Santa Cruz, CA). Cell culture reagents were provided by Gibco (Shanghai, China).

### 2.2. Cell culture

A549 cells and H460 cells, both are established human lung cancer lines, were cultured in fetal bovine serum (FBS, 10%)-containing RPMI medium [18]. BEAS-2B normal lung epithelial cells [19] were obtained from the Cell Bank of Fudan University (Shanghai, China). Cells were maintained in DMEM medium plus 10% FBS. Human HL-7702 hepatocytes were gifts from Dr. Liu’s group [20,21], and hepatocytes were cultured as described previously [20,21].

### 2.3. Culture of patient-derived primary human lung cancer cells

The experiment protocols requiring human specimens were approval by the Ethics Committee of Guangxi University of Traditional Chinese Medicine and in accordance with the Declaration of Helsinki. Two enrolled lung cancer patients (42/56 years old, both male, NSCLC, Stage II) were written-informed. The written consent form to participate in the study was also obtained from the two patients. Surgery-isolated lung cancer specimen was immediately dissected with scalpels. The tumor tissues were then placed in triple enzyme medium (1× collagenase, 1× hyaluronidase, and 1× DNase) in HBSS solution at room temperature for 2–3 hours [22]. Afterwards, most of the solid tumor tissues were dissociated. The resolving cells were filtered through a 70-μm nylon cell strainer (Becton Dickinson, Shanghai, China) and suspended in RPMI 1640 with 10% of FBS.

### 2.4. Methylthiazol tetrazolium (MTT) assay of cell viability

Cells (1×10^4^ cells/well) were seeded onto 96-well plates. Following applied treatment, MTT solution (25 μL/well, 5 mg/mL) was added to each well. After 2-hour incubation, DMSO (200 μL/well, Sigma) was added to dissolve the crystals. The plate was allowed to stand for 10 min, and the optic density (OD) absorbance at 590 nm was recorded. OD values of treatment groups were always normalized to that of untreated control.

### 2.5. Lactate dehydrogenase (LDH) assay

LDH content in the conditional medium indicates the level of cell death. After applied treatment of cells, medium LDH was assayed by a LDH detection kit from Roche Applied Science (Shanghai, China). LDH release % = LDH released in conditional medium/(LDH released in conditional medium + LDH in cell lysates) x 100%.

### 2.6. Clonogenic assay

Two days following applied ONC201 treatment, cells (0.5×10^4^/dish) were detached and re-suspended in 1 mL of medium plus 0.5% agar (Sigma). The mix was then plated onto a pre-solidified 100-mm Petri dish. After 10 days of incubation, the colonies were stained with crystal blue and manually counted.

### 2.7. Sub-G1 analysis of cell apoptosis

Following the applied treatment, lung cancer cells were detached and fixed in 70% ethanol at 4°C at 30 min, which were then stained with propidium iodide (PI). Cells were then analyzed on a Beckman Coulter flow cytometer. Sub-G1 cells were recorded as apoptotic cells.

### 2.8. Single-stranded DNA (ssDNA) ELISA assay of apoptosis

Denatured ssDNA, a characteristic marker of cell apoptosis, was detected through a nucleosomal monoclonal antibody in an ELISA format. Briefly, cells (1×10^4^ cells/well) were seeded onto 96-well plates. After applied treatment, cell apoptosis was analyzed via the ssDNA ELISA kit (Chemicon, Shanghai, China) according to the protocol attached. The OD value (at 490 nm) was measured as a quantitative indicator of cell apoptosis.

### 2.9. Caspase activity assay

After treatment of cells, cytosolic proteins (30 μg lysates per treatment) were added to the caspase assay buffer (312.5 mM HEPES, pH 7.5, 31.25% sucrose, 0.3125% CHAPS) with Ac-DEVD-AFC (10 μg/mL, CalBiochem) as the caspase-3 substrate, or Ac-IETD-AFC (10 μg/mL, CalBiochem) as the caspase-8 substrate. After incubation for 1 hour under the dark, the released AFC was measured via a spectrofluorometer with excitation of 400 nm [23]. The OD value of treatment group was expressed as the fold change of untreated control group.

### 2.10. Real-time quantitative PCR assay

Total RNA was extracted via the Trizol reagents (Invitrogen, Shanghai, China), and cDNA was synthesized with Superscript VILO cDNA synthesis kit (Invitrogen, Carlsbad, CA). SYBR Green and ABI Prism 7000 equipment (Applied Biosystems, Shanghai, China) were utilized for real-time quantitative PCR assay (RT-qPCR). The primers for human *TRAIL*: forward, 5’-CCTGGGCGATAAAGTGAGAT-3’ and reverse, 5’-GGCCCAGCTGTATGTTGTCT-3’ [24]. 3′. For human *death receptor-5* (*DR5*): forward, 5′-AAGACCCTTGTGCTCGTTGT-3′; and reverse, 5′-AGGTGGACACAATCCCTCTG-3′ [25]. For GAPDH: forward, 5′-CATGAGAA GTATGACAACAGCCT-3′; and reverse, 5′-AGTCCTTCCACGATACCAAAGT-3′ [25]. After amplification, melt curve analysis was performed to analyze product melting temperature. GAPDH gene was chosen as the reference gene for normalization, and the 2^−∆∆Ct^ method was applied to quantify targeted mRNA change within samples.

### 2.11. Western blot assay

Aliquots of 30 μg cell lysates or tumor tissue lysates per treatment were electrophoresed on 10% SDS-PAGE gel, and proteins were transferred to a PVDF (polyvinylidene fluoride) membrane. After transfer, primary and secondary antibody incubations were performed, and the signal was detected by an Enhanced Chemiluminescent detection kit, followed by autoradiography. For detection of nuclear proteins, the nuclei of cultured RPE cells were isolated by the nuclei Isolation kit purchased from Sigma [26].

### 2.12. siRNA transfection

siRNA [scramble control, DR5 (Santa Cruz Biotechnology) or TRAIL (Dharmacon)] transfection of cells was performed with Opti-MEM and Lipofectamine RNAiMAX (Invitrogen, Shanghai, China) using media without antibiotics. After siRNA incubation for 36 hours, expression of DR5 and TRAIL in the transfected cells was tested by Western blot assay.

### 2.13. Mice xenograft tumor assay

For subcutaneous xenografts, 4-/5-week-old female severe combined immunodeficient (SCID) mice were utilized. A549 cells (two millions cells per mouse, in 200-μL suspension of 1:1 Matrigel and PBS) were subcutaneously injected into the right flanks of the mice. All subcutaneous tumors were allowed to reach a detectable volume (∼100–125 mm^3^) before initiating ONC201 treatment. Upon tumor formation, mice (n = 10 per group) were administered either vehicle (saline) or ONC201 at 10 or 50 mg/kg [intraperitoneally (*i*.*p*.)] at day 1, 2, 3, 7, 14, and 21. Tumor volume (in mm^3^), recorded every week, was calculated by the formula: volume = (width)2×length/2. Daily tumor growth was calculated by the tumor volume of the last day (day-35) subtracted that of day-1, which was then divided by the number of days (35). The clinical signs of mice were recorded daily. Humane endpoints were considered as rapid weight loss (>15%), abnormal changes in behavior and motion (social and eating behavior), tumor size greater than 2 cm^3^ or skin problems (wounds or signs of inflammation). The mice were observed extremely carefully throughout the experimental period. If animals reached these endpoints they were euthanized by exsanguination under 2,2,2-tribromoethanol anesthesia (4 mg/10 g body weight, Sigma). All injections in this study were performed via the above anesthesia method [27]. All animal procedures were approved by the Institutional Animal Care and Use Committee (IACUC) and Ethics Committee of Guangxi University of Traditional Chinese Medicine. No mice were dead in the period of experiments.

Tumors were harvested from euthanized mice and homogenized in lysis buffer for Western blot analysis or fixed in 4% paraformaldehyde in PBS for immunohistochemistry (IHC) assay. The staining was performed on cryostat sections (4 μm) of xenograft tissues via the standard methods [28]. Briefly, the slides were incubated with primary antibody (p-Akt Ser473, 1: 50, Cellular Signaling Tech) for 60 minutes, and horseradish peroxidase (HRP)-conjugated secondary antibody for 15 minutes. DAB was applied to stain the antibody positive regions.

### 2.14. Statistical analysis

Results were presented as the mean ± standard deviation (SD) of data from three or more independent experiments. Statistical differences were analyzed by one-way ANOVA followed by multiple comparisons performed with post hoc Bonferroni test (SPSS 20.0). Values of ***p*** <0.05 were considered statistically significant.

## Results

### 3.1. ONC201 induces death in human lung cancer cells

**Fig 1:**
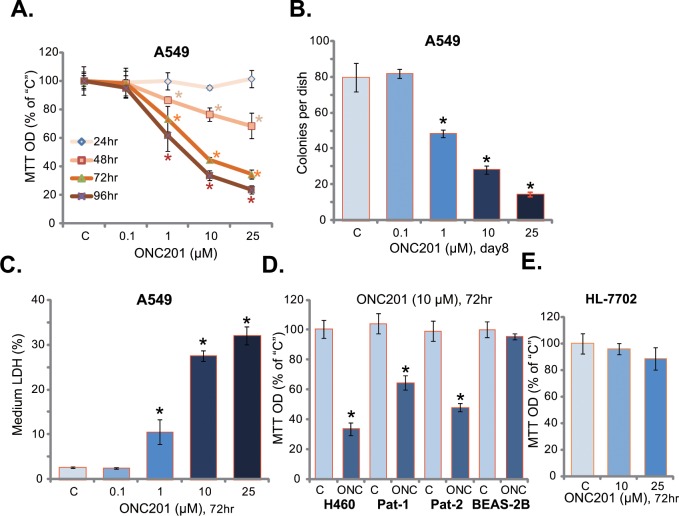
ONC201 induces death in human lung cancer cells. A549 (A-C), H460 (D) or primary human lung cancer cells (“Pat-1/-2”) (D), as well as the lung epithelial BEAS-2B cells (D) and human HL-7702 hepatocytes (E) were treated with applied concentration of ONC201 for indicated time, cells were subjected to MTT assay (A, D and E), colony formation assay (B) and LDH release assay (C). The results presented were representative of three independent experiments. The values were expressed as the means ± SD. “C” stands for untreated control group. *p<0.05 vs “C” group.

First, we examined the potential function of ONC201 on A549 lung cancer cells. Routine MTT assay was performed to test cell survival following ONC201 treatment. As demonstrated in Fig 1A, ONC201 efficiently inhibited A549 cell survival. The anti-survival activity of ONC201 was apparently dose-dependent (Fig 1A). Meanwhile, a time-dependent response by ONC201 was also noticed (Fig 1A). It took at least 48 hours for ONC201 (1–25 μM) to exert a significant cytotoxic effect (Fig 1A). Clonogenicity assay results in Fig 1B demonstrated that ONC201 decreased the number of viable A549 colonies. Meanwhile, the content of medium LDH was significantly increased in ONC201 (at 1–25 μM)-treated A549 cells (Fig 1C). These results confirmed the cytotoxic effect of ONC201 against A549 cells.

The potential effect of ONC201 in other lung cancer cells was also analyzed. In both established (H460 cell line) and primary (patient-derived) human lung cancer cells, treatment with ONC201 (10 μM) again inhibited cell survival (Fig 1D). Note that two primary human lung cancer cell lines (“Pat-1/Pat-2”) were established in the study, each was targeted by ONC201 (Fig 1D). On the other hand, the same ONC201 treatment was non-cytotoxic to human lung epithelial cells (BEAS-2B) [19] (Fig 1D) nor to human HL-7702 hepatocytes (Fig 1E). Together, these results showed that ONC201 was selectively cytotoxic to human lung cancer cells.

### 3.2. ONC201 provokes apoptosis in human lung cancer cells

**Fig 2:**
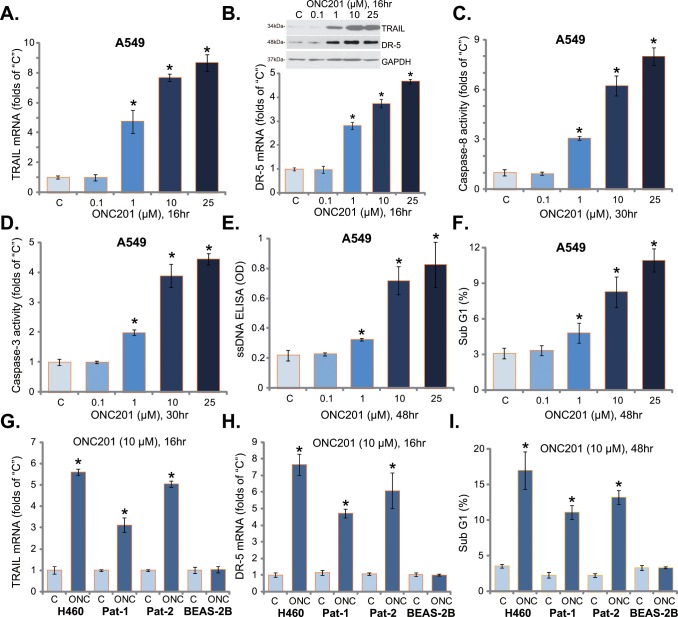
ONC201 provokes apoptosis in human lung cancer cells. A549 (A-F), H460 (G-I) or primary human lung cancer cells (“Pat-1/-2”) (G-I), as well as the lung epithelial BEAS-2B cells (G-I) were treated with applied concentration of ONC201 for indicated time, TRAIL and DR5 expressions were tested by real-time PCR assay (A, B, G and H) or Western blot assay (B, Upper panel); Relative caspase-8/-3 activity was also presented (C and D); Cell apoptosis was tested by ssDNA ELISA assay (E) and Sub-G1 FACS assay (F and I). The results presented were representative of three independent experiments. The values were expressed as the means ± SD. “C” stands for untreated control group. *p<0.05 vs “C” group.

ONC201 is shown to induce TRAIL/DR5 expression and to provoke extrinsic apoptosis pathway activation [12,13]. We therefore tested these signaling events in ONC201-treated lung cancer cells. Real-time quantitative PCR assay was performed, and results showed that ONC201 dose-dependently increased mRNA expression of TRAIL (Fig 2A) and DR5 (Fig 2B, Lower panel). Meanwhile, the protein expression of TRAIL and DR5 was also unregulated in ONC201-treated cells (Fig 2B, Upper panel). Further, the caspase-8 activity was increased following ONC201 treatment in A549 cells (Fig 2C).

Since caspase-8 activation and TRAIL expression are characteristic markers of extrinsic apoptosis pathway activation, we next tested apoptosis activation in ONC-treated lung cancer cells. Results showed that ONC201 treatment in A549 cells dose-dependently increased caspase-3 activity (Fig 2D), ssDNA apoptosis ELISA OD (Fig 2E) and percentage of Sub G1 phase cells (Fig 2F), indicating apoptosis activation. In H460 and primary human lung cancer cells, treatment with ONC201 (10 μM) similarly induced TRAIL (Fig 2G) and DR5 (Fig 2H) expression, as well as apoptosis activation (Fig 2I). Intriguingly, same ONC201 treatment again failed to induce above apoptosis effects in BEAS-2B lung epithelial cells (Fig 2G–2I). These results suggest that ONC201 provokes extrinsic apoptosis pathway activation in human lung cancer cells.

### 3.3. Inhibition of extrinsic apoptosis activation attenuates ONC201’s cytotoxicity in human lung cancer cells

**Fig 3:**
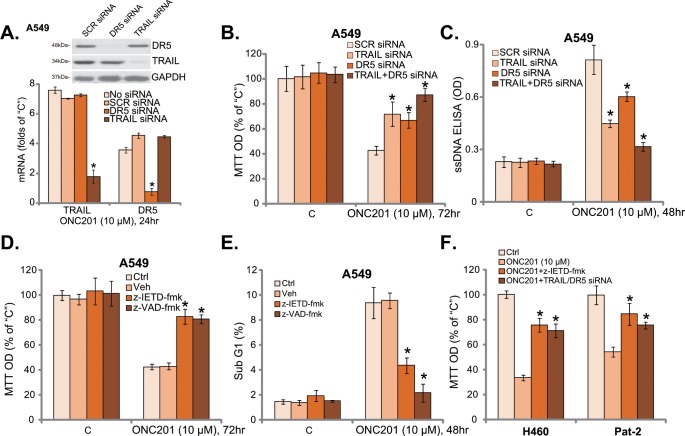
Inhibition of extrinsic apoptosis activation attenuates ONC201’s cytotoxicity in human lung cancer cells. A549 cells transfected with scramble control siRNA (SCR-siRNA), TRAIL siRNA or DR5 siRNA (100 nM each) were treated with ONC201 (10 μM) for applied time, TRAIL/DR5 mRNA and protein expressions were shown (A, GAPDH was tested as the control); Cell viability and apoptosis were examined by MTT assay (B) and ssDNA apoptosis ELISA assay (C), respectively. A549 cells were pre-treated with z-IETD-fmk (40 μM) or z-VAD-fmk (40 μM) for 1 hour, followed by ONC201 (10 μM) treatment for indicated time, cell viability and apoptosis were examined by MTT assay (D) and Sub G1 FACS assay (E), respectively. H460 cells or primary human lung cancer cells (“Pat-2”), pretreated with z-IETD-fmk (40 μM, 1 hour) or TRAIL plus DR5 siRNA (100 nM each, 36 hours), were treated with ONC201 (10 μM) for 72 hours, cell viability was tested by MTT assay (F). The results presented were representative of three independent experiments. The values were expressed as the means ± SD. “C” stands for untreated control group. “Veh” stands for 0.2% DMSO (D and E). *p<0.05 vs “No siRNA” group (A). *p<0.05 vs “SCR siRNA” group (B and C). *p<0.05 vs “Veh” group (D and E). *p<0.05 vs ONC201 only group (F).

The above results showed that ONC201 induced death and activated extrinsic apoptosis pathway in human lung cancer cells. We next tested the link between the two. First, siRNA strategy was applied to knockdown TRAIL and DR5. RT-qPCR and Western blot assay results in Fig 3A showed that expression of TRAIL or DR5 was largely inhibited by the targeted siRNA in A549 cells. Consequently, ONC201-induced cytotoxicity (Fig 3B) and apoptosis (Fig 3C) were attenuated with TRAIL or DR5 siRNA knockdown. Intriguingly, knockdown of both TRAIL and DR5 led to a more profound inhibition on ONC201’s cytotoxicity (Fig 3B and 3C).

We next utilized the caspase-8 specific inhibitor z-IETD-fmk and the pan caspase inhibitor z-VAD-fmk. Results showed that both z-IETD-fmk and z-VAD-fmk significantly attenuated ONC201-induced survival loss (Fig 3D) and apoptosis (Fig 3E). Similar results were also obtained in H460 and primary human lung cancer cells (“Pat-2”), where TRAIL plus DR5 siRNA knockdown or caspase-8 inhibition (z-IETD-fmk) significantly alleviated ONC201’s cytotoxicity. There results suggest that ONC201’s cytotoxicity against lung cancer cells requires activation of extrinsic apoptosis pathway.

### 3.4. ONC201 inactivates of Akt-mTOR and Erk, forcing Foxo3a nuclear translocation in lung cancer cells

**Fig 4:**
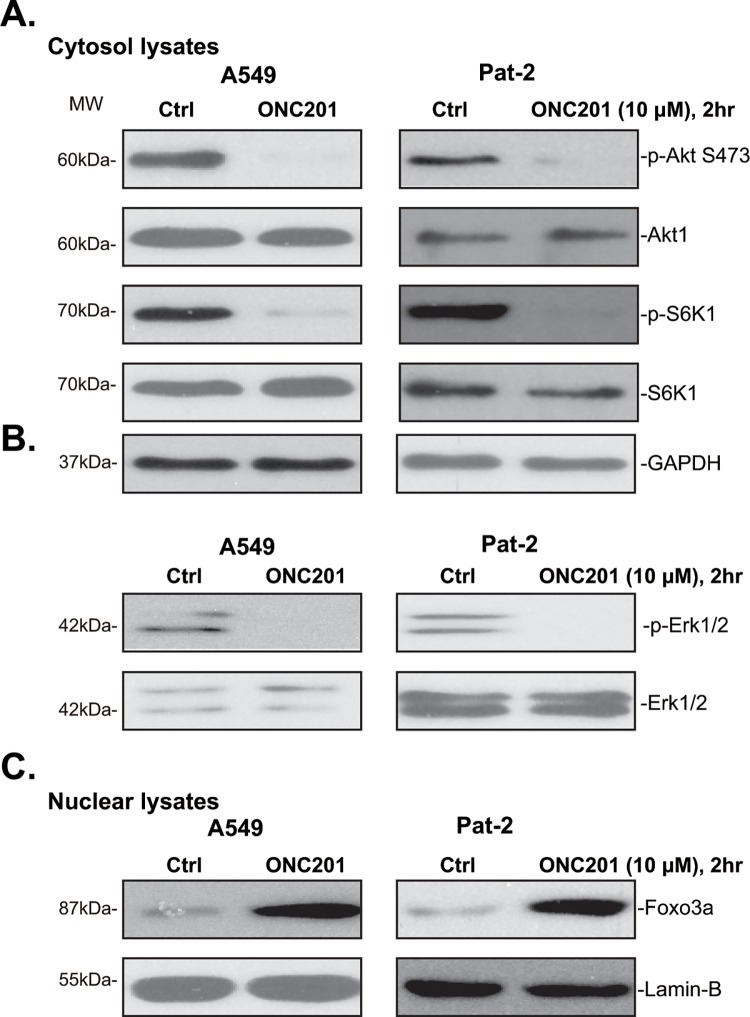
ONC201 inactivates of Akt-mTOR and Erk, forcing Foxo3a nuclear translocation in human lung cancer cells. A549 or primary human lung cancer cells (“Pat-2”) were treated with or without ONC201 (10 μM) for 2 hours, expression of listed proteins in cytosol lysates (A and B) and nuclear lysates (C) was tested by Western blot assay. GAPDH was tested as the control. The results presented were representative of three independent experiments.

Above results showed that ONC201 induced potent cytotoxic and pro-apoptotic activities in human lung cancer cells. Both Akt-mTOR [29,30,31] and Erk [32] signaling cascades are important for lung cancer cell survival and/or apoptosis resistance. Recent studies showed that ONC201 could inhibit above signalings in several different cancer cell lines, which is required for TRAIL/DR5 expression [12,13,17,33]. We therefore examined Akt-mTOR and Erk activation in lung cancer cells with ONC201 treatment. Western blot results in Fig 4A showed that ONC201 dramatically inhibited p-Akt (Ser-473) and p-S6K1 (Thr-389) in A549 cells and primary human lung cancer cells (“Pat-2”), indicating Akt-mTOR inhibition. Further, Erk activation (p-Erk1/2) was also largely inhibited by ONC201 in above lung cancer cells (Fig 4B). We also noticed Foxo3a nuclear translocation following ONC201 treatment in above lung cancer cells (Fig 4C), which reportedly is required for TRAIL and DR5 transcription [13,33]. These results indicate that ONC201 inactivates Akt-mTOR and Erk, therefore forcing Foxo3a nuclear translocation in lung cancer cells. Notably, basal Akt and Erk activation was high in cancer (A549) cells, but was quite low in BEAS-2B lung epithelial cells (S1 Fig, upper panel). That could explain why these cells were not responsible to ONC201: TRAIL and DR5 were not induced by the same ONC201 treatment in these epithelial cells (S1 Fig, lower panel), nor these epithelial cells were killed by ONC201 (Fig 1).

### 3.5. Intraperitoneal injection of ONC201 inhibits A549 xenograft tumor growth in SCID mice

**Fig 5:**
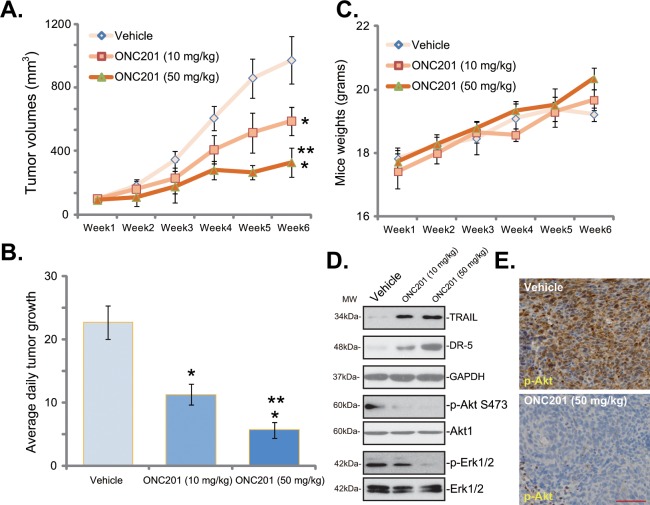
Intraperitoneal injection of ONC201 inhibits A549 xenograft tumor growth in SCID mice. SCID mice bearing A549 tumors (n = 10) were administrated with vehicle control (“Saline”), or ONC201 (10/50 mg/kg, *i*.*p*.), tumor volumes (in mm^3^, A) and mice body weights (in grams, C) were recorded every week for a total of 5 weeks. The estimated daily tumor growth (in mm^3^ per day, B) was also presented. The signaling molecule in the xenografted tumor tissues (3 days post initial ONC201 treatment) were tested by Western blot assay (D) and IHC staining (E, for p-Akt). * ***p*** < 0.05 vs. group of Vehicle control. ** ***p*** < 0.05 vs. group of ONC201 at 10 mg/kg. The above xenograft experiments were repeated twice, and similar results were obtained. Bar = 100 μm (E).

At last, the *in vivo* activity of ONC201 was evaluated. As described, a sufficient number of A549 cells were inoculated into the right flanks of SCID mice to establish xenograft A549 tumors. Intraperitoneal injection of ONC201 at 10 mg/kg or 50 mg/kg potently inhibited A549 tumor growth *in vivo* (Fig 5A). ONC201’s anti-tumor activity appeared dose-dependent, and 50 mg/kg of ONC201 was more potent than 10 mg/kg in inhibiting A549 tumor growth (Fig 5A). As shown in Fig 5B, the estimated daily tumor growth volume of ONC201-treated mice was significantly lower than that of vehicle control mice (Fig 5B). Notably, the mice body weight was not significantly affected by the ONC201 treatment (Fig 5C), suggesting that mice were well-tolerated to the ONC201 regimens. When analyzing tumor tissue samples via Western blot assay, we showed TRAIL-DR5 expression and Akt-Erk in-activation in ONC201-treated tumors (Fig 5D). Meanwhile, IHC image results in Fig 5E confirmed Akt inhibition in ONC201-treated A549 tumors.

## Discussions and Conclusion

ONC201 is entering clinical trials due to its favorable preclinical profile that includes robust anti-tumor activity in several preclinical cancer studies [14,15,16]. It has displayed promising anti-cancer results *in vitro* and *in vivo*. For example, Ishizawa et al., have displayed the clinical potential of TIC10 in hematological malignancies [15]. It was shown that ONC201 induced integrated stress responses (ISR) and apoptosis in mantle cell lymphoma (MCL) and acute myeloid leukemia (AML) cells [15]. Meanwhile, Kline et al., demonstrated that ONC201 induced solid tumor cell apoptosis via TRAIL pathway [16].

In the present study, we showed that ONC201 was cytotoxic, anti-proliferative and pro-apoptotic in primary and established (A549/H460) human lung cancer cells. ONC201 inhibited activation of Akt-S6K1 and Erk signalings, leading to Foxo3a nuclear translocation and TRAIL/DR5 transcription in lung cancer cells. siRNA-mediated knockdown of TRAIL and/or DR5 alleviated ONC201’s cytotoxicity against lung cancer cells. For the *in vivo* studies, we showed that intraperitoneal injection of ONC201 at well-tolerated doses significantly inhibited xenografted A549 tumor growth in SCID mice. Above signaling changes were also observed in ONC-201-treated tumors. Therefore, the preclinical results suggest that ONC201 could be further studied as a valuable agent for lung cancer treatment.

Despite inducing a strong cytotoxic effect in tumor cells, ONC201 didn’t induce significant cell death in normal lung epithelial cells in the same conditions. Meanwhile, recent studies have confirmed that ONC201 failed to induce genotoxic effect based on gamma-H2AX assessment [33]. In accordance with *in vitro* profile, efficacious ONC201 doses in murine models were also well tolerated without apparent toxicities or significant loss of body weight. The lack of cytotoxicity of ONC201 to normal cells or tissues was also observed in other studies [33]. We found that ONC201 failed to induce DR5 and TRAIL in normal cells at doses that cause strong DR5 and TRAIL induction in lung cancer cells (Data not shown). One possibility could be that basal Akt and Erk activation was quite low in epithelial cells, and treatment of ONC201 didn’t induce significant Akt and Erk inhibition, therefore no DR5 or TRAIL was induced (Data not shown). In summary, the preclinical study demonstrates that the small molecular TRAIL inducer ONC201 induces potent anti-lung cancer activity both *in vitro* and *in vivo*.

## Supporting Information

S1 Fig A549 and the lung epithelial BEAS-2B cells were treated with or without ONC201 (10 μM) for 2 hours (upper panel) and 16 hours (lower panel), expression of listed proteins was tested by Western blot assay. The results presented were representative of three independent experiments.
(EPS)
